# Standard reference values of the upper body posture in healthy middle-aged female adults in Germany

**DOI:** 10.1038/s41598-021-81879-0

**Published:** 2021-01-27

**Authors:** Daniela Ohlendorf, Polyna Sosnov, Julia Keller, Eileen M. Wanke, Gerhard Oremek, Hanns Ackermann, David A. Groneberg

**Affiliations:** 1 Institute of Occupational Medicine, Social Medicine and Environmental Medicine, Goethe-University, Frankfurt/Main, Theodor-Stern-Kai 7, Building 9A, 60590 Frankfurt/Main, Germany; 2 Institute of Biostatistics and Mathematical Modeling, Goethe-University, Frankfurt/Main, Theodor-Stern-Kai 7, Building 11A, 60596 Frankfurt/Main, Germany

**Keywords:** Biophysics, Physiology, Medical research

## Abstract

In order to classify and analyze the parameters of upper body posture, a baseline in form of standard values is demanded. To this date, standard values have only been published for healthy young women. Data for female adults between 51 and 60 years are lacking. 101 symptom-free female volunteers aged 51–60 (55.16 ± 2.89) years. The mean height of the volunteers was 1.66 ± 0.62 m, with a mean body weight of 69.3 ± 11.88 kg and an average BMI of 25.02 ± 4.55 kg/m^2^. By means of video raster stereography, a 3D-scan of the upper back surface was measured in a habitual standing position. The confidence interval, tolerance range and ICCs were calculated for all parameters. The habitual standing position is almost symmetrical in the frontal plane the most prominent deviation being a slightly more ventral position of the left shoulder blade in comparison to the right. The upper body (spine position) is inclined ventrally with a minor tilt to the left. In the sagittal plane, the kyphosis angle of the thoracic spine is greater than the lordosis angle of the lumbar spine. The pelvis is virtually evenly balanced with deviations from an ideal position falling under the measurement error margin of 1 mm/1°. There were also BMI influenced postural variations in the sagittal plane and shoulder distance. The ICCs are calculated from three repeated measurements and all parameters can be classified as "almost perfect". Deflections from an ideally symmetric spinal alignment in women aged 51–60 years are small-scaled, with a minimal frontal-left inclination and accentuated sigmoidal shape of the spine. Postural parameters presented in this survey allow for comparisons with other studies as well as the evaluation of clinical diagnostics and applications.

## Introduction

With the widespread high standards of living, the impact of demographic change on societies has become increasingly noticeable^1^. The average age of populations and the percentage of older people is rising^2^, a high number of whom are healthy and require no assistance in their daily life which is reflected in the disability-free life expectancy^3^. Furthermore, an increasing number of people are working beyond their statutory retirement age^4^.

Nevertheless, while changes in the body posture occur constantly with age, there is currently no adequate classification system for postural parameters. The quantity and quality of transformations need to be evaluated to differentiate the physiological from the pathological processes^5^. Additionally, standardized baselines enable the tracking of the temporal progress of ailments^6^ and therapeutic progress, providing a guideline for judging the necessity for therapy and evaluating its effectiveness^7^.

In many cases, measurements are taken after the reporting of symptoms as back pain, restriction of movement^8^ or visible asymmetries. Therefore, most of the available data regarding the upper body posture originates in medical diagnostics and, thus, deals with patients and a variety of illnesses^9^ or conditions after treatment^10^. Due to these circumstances, the timing of the diagnostics and intervention can occur after the optimum time. With appropriate essential criteria, not only can invasive interventions be avoided, but also risk assessments can be developed which allow the estimation of disease progression or stagnation. Prophylactic procedures, according to the individual risk, can be established which may influence the speed of progression. Consequently, keystones for health-associated parameters^6^ and quality indicators are needed, as, for example, falling is the most frequent cause of fractures and head injuries in older people^11^, while there are indications of a link between the shift of the center of gravity and spinal imbalance^12^.

Although the measured value of the parameter and its classification are important for diagnostics, the speed of temporal evolvement can also be crucial in deciding the need for therapy ^13^. Most of the current literature which concerns the upper body posture in women deals with changes during or after pregnancy^14,15^, following breast cancer treatment^16^ or with participants suffering from osteoporosis^17^, back pain^18^ or other musculoskeletal or degenerative illnesses^19^.

In order to interpret these measurements, references from healthy subjects are needed; these would provide a baseline for further studies and the evaluation of the current status in patients. Reference values can also increase the quality of medical diagnostics by aiding in the choice of the most appropriate therapy and the documentation of its course. It is, therefore, required that reliable and reproducible test procedures are established and that the data on standard values for upper body postures is accessible in order to investigate asymptomatic individuals. With this approach, combining screening tests and individual risk factor analysis together with the raising of awareness of spinal health, the sustainability and quality of medical treatments can be raised, maintaining a high quality of life in all age groups and avoiding the deterioration^12,13^ of posture. One method to visualize the upper body posture without X-ray procedures is video raster stereography.

Video raster stereography is a radiation-free and touch-free method to depict the back surface in three dimensions with a high (intra- and inter-day) reliability and reproducibility^20,21^; with the use of given anatomical landmarks its accuracy increases^22^. Furthermore, the brief procedure simplifies the survey and makes it more accessible for participants^23,24^.

A methodology paper by Ohlendorf et al. ^25^ described the project to measure the upper body posture in dependence of age and gender of the working population in Germany via video raster stereography.

However, up to now, the only published standard values of the upper body posture describe females aged 21–30 years^26^, males aged 18–35 years^27^ and 41–50 years^28^.

Not only age that affects physical changes, but also gender. Gender, for example, has a different life expectancy^29^ and incidence of age-related diseases^30^. With regard to this, women between the ages of 51–60 years are particularly interesting, as menopause usually begins in this age group^31^. Those women experience a special, far-reaching physical change long after the completion of growth and hormonal changes during puberty. Hormonal changes during menopause can lead to postmenopausal osteoporosis^32^ which in turn affects the musculoskeletal system^33^. The spine is often affected by kyphosis or load fractures of the vertebral bodies as a result of bone density loss^34^. Since the menstrual cycle affects postural stability, e.g., through increased parasympathetic activity^35^, its change during or absence after menopause can also affect posture. In addition, with increasing age, muscle mass and physical strength decrease with increasing age^36^. Hormonal changes^37–40^ with increased androgen levels, e.g., testosterone, compared to estrogen levels, may contribute to increased total body fat mass but also to a shift in its distribution^41^. There is also evidence for a link between hormonal balance, the reduction of leg fat and the accumulation of abdominal and visceral fat tissue^42–44^. As the menstrual cycle has an impact on postural stability^35^, its change and absence during the menopause may also affect posture. Conclusively this situation demands an upper body posture classification with the previously addressed scopes of application.

The advantage in surveying a homogenous group lies in reducing the variance and increasing the informative value by minimizing effects of age and gender on postural characteristics.

Thus, the aim of this study is defining these reference values with tolerance range and confidence intervals for healthy women aged 51–60 years. This can provide a baseline for categorization and comparison either for other studies or in clinical application.

## Material and methods

### Subjects

101 physically healthy female volunteers in this study were recruited with ages ranging from 51 to 60 years (55.16 ± 2.89 years). The median height was 1.66 ± 0.62 m (lower tolerance 1.54 m and upper tolerance 1.78 m; lower confidence 1.65 m and upper confidence 1.68 m), the mean weight was 69.3 ± 11.88 kg (lower tolerance 45.02 kg and upper tolerance 93.57 kg; lower confidence 66.9 kg and upper confidence 71.69 kg) and the mean BMI was 25.02 ± 4.55 kg/m^2^ (lower tolerance 15.91 kg/m^2^ and upper tolerance 34.12 kg/m^2^; lower confidence 24.12 kg/m^2^ and upper confidence 25.91 kg/m^2^).

According to the WHO classification^45^, 3.96% were underweight, 52.48% had normal weight, 29.7% were pre-obese and 13.86% were obese. The volunteers were acquired through personal approach in dental offices in Frankfurt am Main (Germany) and Heidelberg (Germany), as well as at the “Carolinum” dental university hospital, Frankfurt am Main (Germany).

All subjects filled out the anamnesis questionnaire of the Centre for Dental, Oral and Maxillofacial Medicine of the Goethe University Frankfurt am Main^46^. In addition to questions regarding TMD (temporomandibular joint dysfunctions) or problems with masticatory muscles, the health status, questions on general diseases such as osteoporosis or diabetes mellitus or weekly physical activity were also asked.

The frequency of their physical activity was surveyed; 76.24% exercised regularly, at least once weekly. Furthermore, the participants had a wide variety of employment, such as working in IT, nurses, teachers, housewives, working in retail or journalists.

All subjects were healthy and free of musculoskeletal complaints and, therefore, they were not being treated for any conditions. Using a questionnaire, disorders in the musculoskeletal or the temporomandibular system were excluded^46^.

Exclusion criteria were: disorders of the musculoskeletal system which required medication, physiotherapy, osteopathic or orthopedic treatment, or were associated with restrictions of movement, e.g. osteoporosis or spinal disc prolapse. In addition, participants who had traumas or operations in the last two years were not included in the study.

A correlation coefficient of ≥ 0.25 (evaluation according to Evans^47^) and a power of 80% showing at least a significant weak correlation can be assumed, hence a case number of n = 100 can be expected. All volunteers were healthy (none were patients who were undergoing medical treatment) and informed about the study design before giving written informed consent. The study was approved by the local medical ethics committee of the medical faculty (Goethe-University Frankfurt; No. 303/16) in accordance with the relevant guidelines and regulations and its later amendments (Declaration of Helsinki, 1964).

### Measurement system

**Figure 1:**
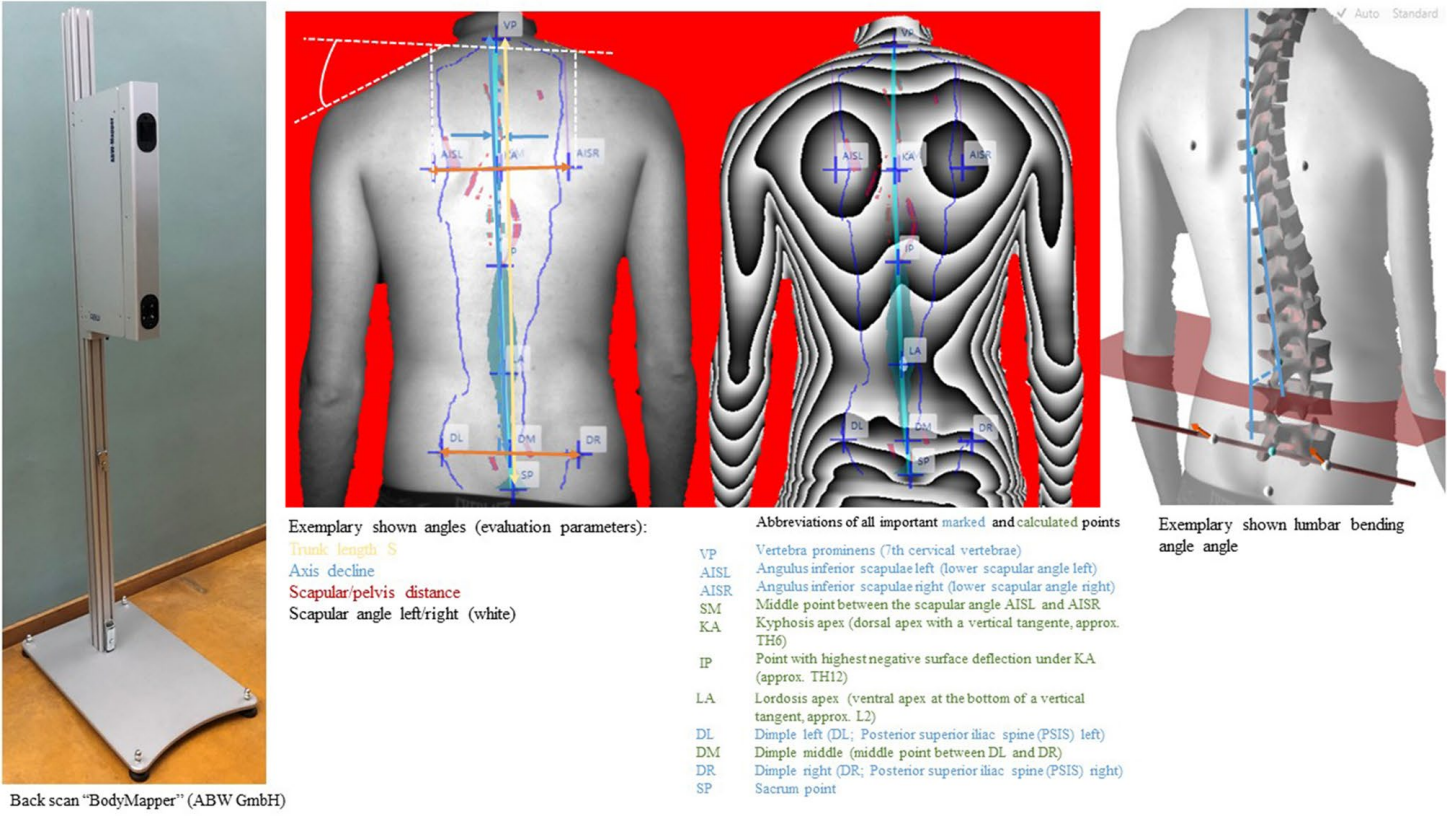
Display of the BodyMapper, drawn exemplary angles, descriptions of marked and calculated points as well as the fictitious calculation of the lumbar bending angle.

In order to determine the upper body posture a three dimensional back scan was conducted with the videorasterstereography back mapper "ABW-BodyMapper" (ABW GmbH, Frickenhausen, Germany) (Fig. 1). In the process a defined stripe pattern is projected onto the back surface which is then captured by a camera with a defined angle. The projection has a frequency of 50 Hz and a resolution of 1/100 mm. Through a triangulation technique, a 3D model of the back is obtained and parameters defining the spinal posture are calculated. The scan takes 15 recordings in approximately half a second. The system error is specified as < 1 mm (manufacturer information) and the reproducibility is limited by the calculations of the upper body posture defined by markers directly on the skin (< 0.5 mm). Yi et al.^20^ have calculated the intra- and inter-reliabilies of this measurement system and described it as good in both cases. Furthermore, they also proved the correlation between the Cobb angle via X-ray radiography and the bodymapper of the lordosis and kyphosis angles. They concluded that the accuracy of the data increases with the experience of the investigator who places the landmarks on the back of the subject to be measured. Therefore, an experienced or trained examiner was used for this study.

Twenty-three parameters could be evaluated, grouped into three sections:

The first group describes the shoulder area, the second outlines the spine while the third group characterizes the pelvis (Fig. 1). A detailed description of the parameters can be found in Ohlendorf et al.’s^25^ methods paper.

### Experimental setup

Subjects were asked to undress their upper body down to their underwear, from the neck down to the lower spine; long hair was tied up and necklaces or other reflective jewelry was removed. In order to standardize the position, an orientation line was placed orthogonally to the scanner on the floor to align the big toes. Six reflective markers were then placed on the skin surface, according to the following defined skeletal structures: vertebrae prominens C7, sacrum point at the beginning of the intergluteal cleft, angulus inferior scapulae left and right and dimple left and right^25^. Light in the room was dimmed to exclude interference with the scan. Participants were asked to stand in their habitual position, with their arms hanging loosely beside their torso and their line of vision directed straight ahead. Three back scans were performed sequentially to minimize intra-individual measuring errors.

Three repeat measurements were taken within 2 min and subsequently averaged.

### Statistics

All calculations were performed with Bias 11.08 (Epsilon-Verlag, Darmstadt, Germany). The normal distribution of each parameter was tested by using the Kolmogorov–Smirnov-Test with Lilliefors-correction. Mean and median values were calculated accordingly. Upper and lower limits of the tolerance area (TA) show the normal range with 95% of all values within ± 2SD of the average. In order to determine the range of the mean or median values, a 95% confidence interval (CI) was computed; these data were calculated according to the parametric or non-parametric distribution of the parameters.

Since there was a great heterogeneity of the test persons regarding the BMI, a BMI group comparison was carried out using the Kruskal–Wallis test, followed by a multiple Conover-Iman comparison including Bonferroni-Holm correction of the p-values. The same test procedure was used to compare the sporting activity of the subjects. The significance level was 5%.

Further, Intra-class correlations (ICC) were calculated for all parameters, since three repeated measures were collected for each subject. All ICCs were classified by means of Landis and Koch suggested ^48^: 0–0.20 = “slight”, 0.21–0.40 = “fair”, 0.41–0.60 = “moderate”, 0.61–0.80 = “substantial”, 0.81–1.00 = “(almost) perfect”.

### Ethics approval and consent to participate

This study was approved by the Ethics Committee (303/16) of the Goethe University Frankfurt am Main. All participants signed an informed consent to participate in advance.

### Consent to publish

All individuals have given their consent to publish their images.

## Results

Table 1 includes the mean or median values, as well as the tolerance area, confidence interval and Intra-class-correlation of the three measurement receptions of all parameters.

**Table 1:** Upper body posture parameters including the mean or median values, tolerance range, confidence interval, standard deviation and 2 standard deviation. A description of the parameters can be found directly below each respective parameter. Normal distributed data are in italics. ICCs were classified as follows: 0–0.20 = “slight”, 0.21–0.40 = “fair”, 0.41–0.60 = “moderate”, 0.61–0.80 = “substantial”, 0.81–1.00 = “(almost) perfect”.

Parameter	Mean value/median	Lower tolerance	Upper tolerance	Lower confidence	Upper confidence	SD	2SD	ICC
**Shoulder parameter**								
Scapular distance (mm) Distance between the left (AISL) and the lower right scapular angle (AISR)	*164.06*	*120.94*	*207.17*	*159.80*	*168.31*	*21.56*	*43.11*	*0.935*
Scapular height (°) Height difference between the AISL and AISR points	0.15	− 14.63	14.93	− 1.76	1.69	7.39	14.78	*0.968*
Scapular rotation (°) Rotation of the distance AISL—AISR in the transversal plane	*1.40*	*− 4.96*	*7.77*	*0.78*	*2.03*	*3.18*	*6.36*	*0.972*
Left scapular angle (°) Angle of the compensation line applied from the shoulders to the horizontal. The center of the compensation line is specified vertically above AISL	27.28	4.38	50.18	25.22	28.72	11.45	22.90	*0.881*
Right scapular angle (°) Angle of the compensation line applied from the shoulders to the horizontal. The center of the compensation line is specified vertically above AISR	28.53	12.87	44.19	27.19	29.86	7.83	15.66	*0.814*
**Spine parameter**								
Trunk length D (mm) Spatial distance between the markers C7 and middle of the PSIS-marker	*452.32*	*404.20*	*500.44*	*447.57*	*457.07*	*24.06*	*48.12*	*0.993*
Trunk length S (mm) Spatial distance between the markers at C7 and Rima Ani	*487.62*	*433.70*	*541.54*	*482.30*	*492.94*	*26.96*	*53.92*	*0.993*
Sagittal trunk decline (°) Inclination of the trunk length D marked line from the perpendicular to the sagittal plane	*− 3.95*	*− 10.08*	*2.18*	*− 4.56*	*− 3.35*	*3.07*	*6.13*	*0.975*
Frontal trunk decline (°) Inclination of the trunk length D marked line from the perpendicular to the frontal plane	*− 0.31*	*− 2.98*	*2.36*	*− 0.57*	*− 0.05*	*1.33*	*2.67*	*0.949*
Axis decline (°) Deviation of the line of the area marked by the trunk length D line of the 90° rotated distance between PSIS left and PSIS right	− 0.54	− 6.08	5.00	− 0.97	− 0.21	2.77	5.54	*0.944*
Thoracic bending angle (°) Deviation of the distance C7—kyphosis apex from the perpendicular	*14.51*	*6.29*	*22.72*	*13.69*	*15.32*	*4.11*	*8.22*	*0.953*
Standard deviation lateral deviation (°) Root mean squared deviation of the median line of the distance C7—center of the PSIS marker	3.63	− 0.33	7.59	3.06	3.89	1.98	3.96	*0.897*
Standard deviation rotation (°) Root mean square deviation of surface rotation of the median line (torsion of the spinous processes of the spine)	3.81	− 0.57	8.19	3.32	4.18	2.19	4.38	*0.944*
Kyphosis angle (°) Angle between the upper turning point at C7 and the thoracolumbar inflection point	*60.49*	*26.54*	*94.44*	*57.14*	*63.84*	*16.97*	*33.95*	*0.969*
Lordosis angle (°) Angle between the lower inflection point at the center of the PSIS marker and the thoracolumbar turning point	*52.61*	*20.09*	*85.12*	*49.40*	*55.82*	*16.26*	*32.51*	*0.972*
Lumbar bending angle (°) Deviation of the distance kyphosis apex—lordosis apex from the perpendicular	*14.44*	*6.75*	*22.13*	*13.68*	*15.20*	*3.85*	*7.69*	*0.969*
**Pelvic parameter**								
Pelvic distance (mm) Spatial distance between between the left (PSISL) and right (PSISR) of the pelvis	*92.23*	*66.02*	*118.43*	*89.64*	*94.81*	*13.10*	*26.20*	*0.972*
Pelvic height (°) Decline of the connecting line between PSIS left and PSIS right to the horizontal in the frontal plane in degrees	0.00	− 5.26	5.26	− 0.42	0.30	2.63	5.26	*0.945*
Pelvic torsion (°) PSIS left–PSIS right, twist around the transverse axis calculated from the mutual twisting of the surface normal on the two PSIS	*− 0.72*	*− 10.89*	*9.45*	*− 1.73*	*0.28*	*5.09*	*10.17*	*0.897*
Pelvic rotation (°) Rotation of the distance PSIS left–PSIS right in the transversal plane	*0.77*	*− 5.74*	*7.28*	*0.13*	*1.42*	*3.26*	*6.51*	*0.971*

Data not normally distributed are printed in italics.

The back scan values of the first section were as follow: The scapular angle was calculated in relation to the horizontal plane with the median values of 27.28° (TA 4.38°–50.18°; CI 25.22°–28.72°) for the left shoulder and 28.53° (TA 12.87°–44.19°; CI 27.19°–29.86°) for the right shoulder, which shows an almost symmetrical position in the frontal plane. The distance between the left and right angulus inferior scapulae (AIS) had a mean value of 164.06 mm (TA 120.94–207.94 mm; CI 159.8–168.31 mm), the height difference between these two markers had a median value of 0.15 mm (TA − 14.63 to 14.93 mm; CI − 1.76 to 1.69 mm), which is almost equal. The inclination of the AISL-AISR connecting line in the transversal plane had an angle of 1.4° (TA − 4.96° to 7.77°; CI 0.78°–2.03°) which is also almost equal. This indicates a marginal more ventral position of the left shoulder to the right one.

The second section describes the spinal parameters. The distances between C7 and Rima Ani is 487.62 mm (TA 433.70–541.54 mm; CI 482.3–492.94 mm) and between C7 and the middle between PSIS left and PSIS right was 452.32 mm (TA 404.2–500.44 mm; CI 447.57–457.07 mm); these are in relation to the length of the spine. The sagittal inclination of the upper body was − 3.95° (TA − 10.08° to 2.18°; CI − 4.56° to − 3.35°) and the frontal inclination was − 0.31° (TA − 0.31° to − 2.98°; CI − 0.57° to − 0.05°). Overall, the upper body (spine position) was inclined ventrally with a minor tilt to the left. The axial deviation which measures the difference between the angle C7-middle between PSIS left and PSIS right //PSIS left and PSIS right and 90° amounted to -0.54° (TA − 6.08° to 5.00°; CI − 0.97° to − 0.21°), hence there is virtually no deviation between the angle of the trunk axis to the hip and the square angle. The maximal deviation from the median line had a mean standard deviation of 3.63 mm (TA − 0.33 to 7.59 mm; CI 3.06 to 3.89 mm). This means that the participants had a modest bigger maximal deviation to the left than to the right which aligns with the allover maximal lateral deviation. The surface rotation has a standard deviation of 3.81° (TA − 0.57° to 8.19°; CI 3.32°–4.18°).

The inclination of the thorax was 14.5° (TA 6.29°–22.72°; CI 13.69°–15.32°); this is related to the ventral head position. The lumbar bending angle amounted to 14.44° (TA 6.75°–22.13°; CI 13.68°–15.20°). The kyphotic angle was 60.49° (TA 26.54°–94.44°; CI 57.14°–63.84°) and the lordotic angle was 52.61° (TA 20.09°–85.12°; CI 49.4°–55.82°). Following the spinal curve, it can be seen that the kyphosis angle is greater than the lordosis angle, whereas the thoracic and lumbar angles show, essentially, no difference.

The measurements of the third group describe the pelvic region. The distance between the PSIS left and PSIS right was 92.23 mm (TA 66.02–118.43 mm; CI 89.64–94.81 mm), while the height difference between the PSIS left and PSIS right amounted to 0° (TA − 5.26° to 5.26°; CI − 0.42° to 0.30°). The torsion of the pelvis amounted to − 0.72° (TA − 10.89° to 9.45°; CI − 1.73° to 0.28°) and the rotation of the pelvis to 0.77° (TA − 5.74° to 7.28°; CI 0.13°–1.42°).

Contrary to the shoulder inclination in the frontal plane, the pelvic measurements display an almost symmetrical, even position.

According to the classification of Landis and Koch^48^ all ICCs are to be classified as "almost perfect", since the lowest ICC is found at the right scapula angle with an ICC of 0.814. All other ICCs have better values.

### BMI group comparison

The BMI group comparison according to the WHO classification of the BMI was compared with the following groups: group 1 normal weight, group 2 pre-obese, group 3 obese and group 4 underweight.

Significant group differences were found in the sagittal trunk decline (p ≤ 0.001), scapula distance (p ≤ 0.01), kyphosis and lordosis angle (p ≤ 0.001 and 0.01, respectively) and lumbar flexion angle (p ≤ 0.001).

The multiple pair comparison refers to the following group differences in the individual parameters:

– Sagittal trunk decline (p ≤ 0.02) between groups 1 (− 2.89°) and 2 (− 5.27°) and groups 2 and 3 (− 5.90°).

– Shoulder blade distance: no significance.

– Kyphosis angle (p ≤ 0.001) between group 1 (55.07°) and 3 (76.84°).

– Lordosis angle (p ≤ 0.01) between group 1 (48.70°) and 3 (68.47°).

– Lumbar bending angle (p ≤ 0.02 or 0.001) between groups 1 (12.60°) and 2 (15.21°), groups 1 and 3 (17.72°) and groups 3 and 4 (10.79°).

**Figure 2:**
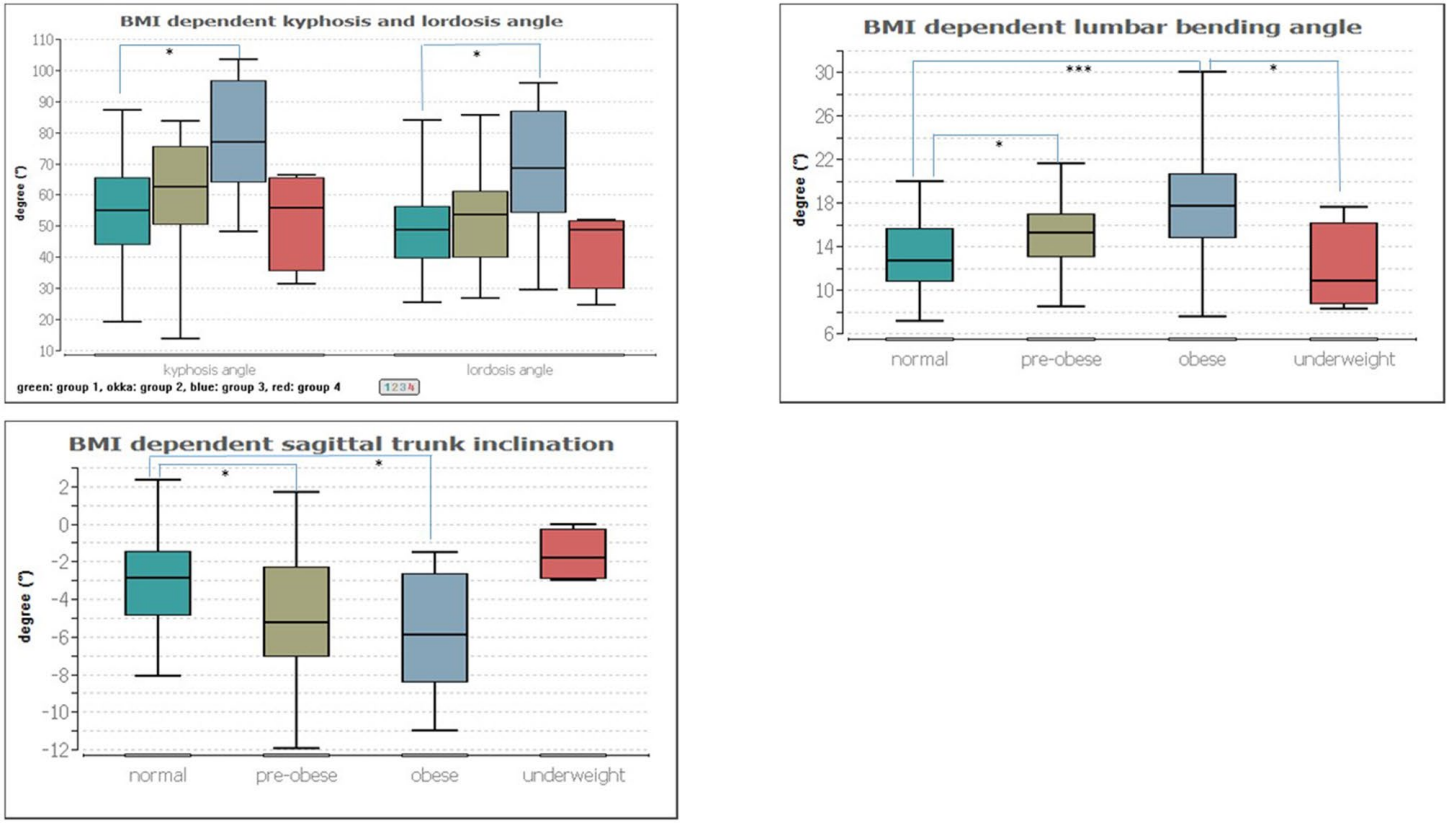
Representation of dependencies between the BMI groups and the kyphosis and lordosis angle (top left), the lumbar flexion angle (top right) and the sagittal trunk inclination. Significant group differences are marked with asterisks, where. * = 0.05, *** = 0.001.

All significant pair comparisons are illustrated in Fig. 2.

### Physical activity

Based on the information they provided in the questionnaire, the women were divided into four groups with regard to physical activity (1: no regular sport, 2: 1x/week, 3: 2x/week, 4: > 2x/week).

The group comparison does not show any significance (p ≥ 0.05). The comparison of subjects who regularly exercise (groups 2, 3 and 4) is also not significant, as is the comparison of active subjects who exercise regularly (n = 26) and those who do not exercise regularly (n = 75) (p ≥ 0,05).

## Discussion

In this paper, standard values for the upper body posture are presented for healthy female subjects aged 51–60 years. Before discussing the reference values, it is useful to assess the anthropometric data. In comparison to the German Mikrozensus (the most important annual household survey of official statistics in Germany) from 2013^49^ and Mensink et al.^50^, the participants in this study were near to the average for the female population^45,49^ especially in height (Table 2). The BMI in the study group was slightly lower than in the German population as there was a higher percentage of underweight and a lower fraction of obese women in the study group. This can be explained by the high percentage (76.26%) of physical activity carried out by the present subjects; 31.68% of the participants reported to exercise three or more times a week which indicates a high awareness of the importance of a healthy lifestyle and a high level of physical activity. Compared to younger women, aged 21–30 years old and also from Germany^26^, the present subjects were smaller (about 3 cm), heavier (about 6 kg) and had a higher BMI (about 3.92 kg/m^2^).

**Table 2:** Comparison of anthropometric parameters between the present results and other studies.

	Present results	German average^49^	Mensink et al. ^50^	Ohlendorf et al.^26^
Age (years)	51–60	51–60	51–60	21–30
Height (cm)	166.00	165.50	163.10	169.00
Weight (kg)	69.30	70.05	73.00	60.30
BMI (kg/m^2^)	25.02	25.55	27.40	21.10
BMI < 18.5 (%)	3.96	2.00	1.00	6.00
BMI 18.5–24.9 (%)	52.48	50.55	38.10	87.80
BMI 25–29.9 (%)	29.70	31.30	33.50	4.70
BMI > 30 (%)	13.86	16.20	27.30	0.90

With regard to the reference values, it should first be noted that the women participating in this study have an almost ideally balanced, or rather symmetrical, upper body posture. Considering the sagittal plane, the posture is kyphotic on a small scale with the kyphosis angle being greater than the lordosis angle by 7.88°. In detail, the participants´ upper body posture has a small rotational component, with the right shoulder having a higher position than the left while also standing more dorsally, but with the pelvic region being balanced; this torsion is limited to the thoracic region. In addition, their spines tilted to the left, on average. The deviations from an ideally symmetrical or balanced position indicated tendencies although these fall mostly within the measurement error range.

One factor that affects posture is the BMI. It is noticeable that with increasing BMI, especially the comparison between normal and obese subjects, the sagittal trunk inclination becomes more pronounced (greater forward inclination in the cervical and thoracic spine area), and the kyphosis angle, the lordosis angle and the lumbar flexion angle increase. Compared to the BMI, the frequency of physical activity has no influence on the upper body posture. An increasing BMI is often associated with more muscle mass and also more surface fat. Since this measurement method is a representation of the back surface, these changes are obvious. The approx. 20° larger lordosis and kyphosis angle in overweight subjects must, however, be analyzed in further studies in a more detailed way.

The comparison of age-related changes in upper body posture compared to the young group of female subjects aged 21–30 years^26^ shows the following differences:

In the shoulder region, the position of the shoulder blades is very similar, except for the distance of the anguli inferior scapula which is wider in the older women by 13.5 mm. The scapular rotation also indicates a slightly further dorsally positioned right shoulder, but to a smaller extent (1.66°). Furthermore, the pelvic rotation is also lower by 1.43°, with the younger women having a mirrored pelvic torsion and overall mirrored rotation. With the last two differences falling under the error margin of 1°, the overall difference is small scaled. Opposed to the shoulder width, the pelvic distance is smaller in the present, older participants by 7.33 mm.

Regarding the spinal column, the axis decline is tilted in the opposite direction by 0.75° which falls under the error margin of 1°. In addition, the older women display a smaller overall rotation by 3.42°, whereas the kyphosis (difference 8.83°) and lordosis (difference of 6.32°) angles are greater, indicating a further curved spine.

There may be several reasons for these differences: a higher mean weight and BMI are observed with increasing age^41^, while a declining muscle mass and strength have also been recorded^36^. These changes could be reinforced by hormonal changes^38^ during and after menopause but may also be mitigated by physical activity^51^. Athletic activity has no influence on the body posture, although on the muscular constitution.

The distinctive hormonal balance in postmenopausal women^39,40^ may lead to an increasing fat mass or rather changes in fat distribution^41^. Hormonal shifts with an increased androgen level, e.g., testosterone in comparison to estradiol may be linked to a lower fat percentage in the legs and an accumulation of abdominal and visceral fat^43^.

Drzał-Grabiec et al.^40^ compared two groups of 130 women of different age decades (group 1:60–90 years, group 2: 20–25 years) in terms of upper body posture. There was an increased thoracic kyphosis in the first group in comparison to the second group, an effect which has also been shown by Kado^52,53^. This effect is often caused by age-related changes in components of the spine, such as a loss of height in intervertebral discs and decreasing elasticity of ligaments. Gong et al. 2019^39^ in a study of 226 subjects aged from 20 to 89 years, demonstrated an increase of cervical lordosis and thoracic kyphosis. It was also shown that differences between the genders decrease with age, especially after the 6^th^ decade in the neck region and after the 7th decade in the thoracic region.

Changes in the lumbar lordosis were more heterogeneous, with ranges from no significant differences between younger and older people^54^ to a decreasing lumbar lordosis^55^.

As Gong et al.^39^ used a broader age span of participants and, thus, fewer women in each age category (12 females aged 20–29 years, 12 aged 30–39 years, 8 aged 40–49 years, 12 aged 50–59 years, 20 aged 60–69 years, 26 aged 70–79 years and 12 aged 80–89 years), their study is more likely to be prone to inter-individual fluctuations affecting the results.

The upper body posture is also an important basis of assessment of life quality^7^. Many age related health problems are tightly interwoven with spinal changes as the shifting of the center of gravity^52^. These changes are often interconnected by compensatory mechanisms, thus the change in one parameter involves the adjustment of another^56^. With the participants of this study having, for example, a more pronounced spinal curvature than the younger group while also being healthy and physically active, the physiological changes of postural parameters with age and their respective classification would allow for assessment and risk analysis. As most professions have a sedentary component, the long-term effects of sitting on the parameters of the spine should be investigated.

Limitations of this study are potential causes of measurement errors and should be taken into consideration; for example lightening conditions, such as highlighted spots due to singular light rays or reflective hair accessories (which should be removed before the scan) and extensive dark areas, such as large tattoos or shadows caused by excessive skin folds, can also affect the measurement process^23^.

In addition, the position of the BodyMapper in relation to the participant, or the placing of the reflective markers, can also influence the outcome. However, in this study, the placing was performed according to a standardized procedure^57^ by trained examiners. Under the premise of an experienced user of the BodyMapper good inter-class correlations can be demonstrated (Table 1). Additionally, Yi et al.^20^ show appropriate intra- and inter-rater-reliabilies. Additional limitations of this study are described and discussed in the preliminary method paper^25,26^.

Further investigations should expand these standard values with data regarding the various sex and age constellations to provide a solid baseline for scientific studies, clinical documentation and therapeutical application. Based on this broad database, it could also be investigated as to how the parameters change with age and sex. According to the influence of sedentary time^32,58^, the proportion of working hours should be investigated further in order to evaluate its effects on posture and links to risk assessment associated with spinal parameters^7,52^.

## Conclusion

Overall, the women participating in this study have a balanced upper body posture with a slight tilt to the left in the shoulder and spinal column, as measured by the video raster stereography. Further studies could amend this data by using groups of other ages and genders to observe differences and parallels. Furthermore, the diagnostics of misalignments in upper body posture and therapeutic progress in the treatment of spinal conditions could be evaluated.

## Data Availability

All relevant data are in the manuscript.

## Abbreviations

TA: Tolerance area
CI: Confidence interval
